# Comparative analysis of serologic cross-reactivity using convalescent sera from filovirus-experimentally infected fruit bats

**DOI:** 10.1038/s41598-019-43156-z

**Published:** 2019-04-30

**Authors:** Amy J. Schuh, Brian R. Amman, Tara S. Sealy, Timothy D. Flietstra, Jonathan C. Guito, Stuart T. Nichol, Jonathan S. Towner

**Affiliations:** 1 Viral Special Pathogens Branch, Division of High-Consequence Pathogens and Pathology, Centers for Disease Control and Prevention, Atlanta, GA 30329 USA; 2 Commissioned Corps, United States Public Health Service, Rockville, MD 20852 USA; 3 Department of Pathology, College of Veterinary Medicine, University of Georgia, Athens, GA 30602 USA

**Keywords:** Infectious-disease diagnostics, Viral reservoirs, Ebola virus, Marburg virus, Antibodies

## Abstract

With the exception of Reston and Bombali viruses, the marburgviruses and ebolaviruses (family *Filoviridae*) cause outbreaks of viral hemorrhagic fever in sub-Saharan Africa. The Egyptian rousette bat (ERB) is a natural reservoir host for the marburgviruses and evidence suggests that bats are also natural reservoirs for the ebolaviruses. Although the search for the natural reservoirs of the ebolaviruses has largely involved serosurveillance of the bat population, there are no validated serological assays to screen bat sera for ebolavirus-specific IgG antibodies. Here, we generate filovirus-specific antisera by prime-boost immunization of groups of captive ERBs with all seven known culturable filoviruses. After validating a system of filovirus-specific indirect ELISAs utilizing infectious-based virus antigens for detection of virus-specific IgG antibodies from bat sera, we assess the level of serological cross-reactivity between the virus-specific antisera and heterologous filovirus antigens. This data is then used to generate a filovirus antibody fingerprint that can predict which of the filovirus species in the system is most antigenically similar to the species responsible for past infection. Our filovirus IgG indirect ELISA system will be a critical tool for identifying bat species with high ebolavirus seroprevalence rates to target for longitudinal studies aimed at establishing natural reservoir host-ebolavirus relationships.

## Introduction

Following spillover from a zoonotic source, the filoviruses can be transmitted person-to-person through direct contact with infectious bodily fluids, resulting in large outbreaks of viral hemorrhagic fever with high case fatality^1^. The family *Filoviridae* includes two genera of viruses that are known to cause disease in humans and/or non-human primates; the genus *Marburgvirus* includes one virus species comprised of two viruses (Marburg virus and Ravn virus) and the genus *Ebolavirus* includes six virus species comprised of one virus each (Ebola virus, Bundibugyo virus, Taï Forest virus, Sudan virus, Reston virus and Bombali virus)^2^. With the exception of Reston^3^ and Bombali viruses^4^, the marburgviruses and ebolaviruses cause outbreaks of viral hemorrhagic fever in sub-Saharan Africa^5–10^. Reston virus causes disease in non-human primates only, and its circulation appears to be limited to the Philippines and China^1^. Far less is known about Bombali virus, as its genomic viral RNA was only recently detected in Angolan free-tailed bats (*Mops condylurus*) and little free-tailed bats (*Chaerephon pumilus*) in Sierra Leone^4^. To date, Bombali virus has not reportedly been isolated.

Since the discovery of Marburg virus in 1967^5^, many of the index cases of marburgvirus disease reported entering bat-inhabited caves or underground mines prior to becoming ill^6,11–13^. Retrospective investigation of three outbreaks of marburgvirus disease among miners working in Goroumbwa Mine in northeastern Democratic Republic of the Congo (DRC) from 1998–2000^14^, miners working in Kitaka Mine in southwest Uganda in 2007^15^, and two tourists that had separately visited the nearby Python Cave, Uganda in 2008^16,17^ revealed that all of these locations were inhabited by large populations of the Egyptian rousette bat (ERB; *Rousettus aegyptiacus*). A longitudinal ecological investigation later identified this bat species as a natural reservoir host for the marburgviruses and a source of virus emergence in humans^18^.

Unlike the spillover events leading to marburgvirus disease, those for ebolavirus disease have been difficult to associate with a specific environment, such as caves. Furthermore, the evidence linking ebolavirus spillover to a particular species of bat is largely circumstantial. For example, the 1976 and 1979 index cases of Sudan virus disease both worked in a cotton factory in Sudan, where a retrospective investigation eight to nine months after the initial outbreak identified a large roof colony of Trevor’s free-tailed bats (*Mops trevori*) directly over the working area of the index case^19^. The putative index case of the 2007 Ebola virus disease outbreak in the Kasaï-Occidental province of the DRC was said to have purchased bats for consumption following a reported annual migration of hammer-headed bats (*Hypsignathus monstrosus*) and Franquet’s epauletted fruit bats (*Epomops franquetti*)^20^. Lastly, the presumed index case of the 2014 Ebola virus disease outbreak that started in Guinea was reported to have played in a tree hollow, where DNA traces of *Mops condylurus* were later identified^21^.

The majority of research directed towards the identification of the natural reservoir hosts of the ebolaviruses has consisted of cross-sectional surveillance of the sub-Saharan African and Asian bat population for evidence of active and past ebolavirus infection. The predominance of cross-sectional studies can be attributed to the large number of bat species residing within the geographic range of ebolavirus circulation that require investigation, as well as the challenges associated with obtaining diagnostic specimens from a mammal that is difficult to capture due to its elusive nocturnal nature and ability to fly. Collectively, Ebola virus RNA has been detected in three bat species captured in the Republic of the Congo and Gabon (*Hypsignathus monstrosus*, *Epomops franquetti* and *Myonycteris torquata*)^22^ and Reston virus RNA has been reportedly detected in four bat species captured in the Philippines (*Chaerephon plicatus*, *Cynopterus brachyotis*, *Miniopterus australis* and *Miniopterus schreibersii*)^23^. Yet, infectious virus was not isolated from any of these species^22,23^, indicating that they are either dead-end virus hosts, had cleared virus infection prior to sampling, or that current filovirus isolation techniques lack the sensitivity to recover infectious virus from specimens with low viral loads. Serological reactivity of bat sera with ebolavirus antigens using indirect ELISAs, indirect fluorescent tests, bead-based multiplex assays and/or western blots has been reported in 375 bats representing at least 21 species throughout sub-Saharan Africa and Asia^22–32^. However, interpretation of this data has been exceedingly difficult due to multiple reasons that include: (1) the absence of a panel of positive and negative bat sera for initial serological assay validation and continuing quality control, (2) the use of various uncharacterized recombinant filovirus antigens, and (3) the application of different statistical methods for establishing cutoff values for seropositivity. Nonetheless, ebolavirus serosurveillance offers several distinct advantages compared to surveillance for active virus infection including a longer diagnostic window (i.e., length of time a diagnostic test can detect evidence of infection), no knowledge of virus-tissue tropism and no requirement for destructive sampling.

Although ERBs are natural reservoir hosts of the marburgviruses only, they develop a robust virus-specific IgG antibody response following experimental inoculation with the ebolaviruses^33,34^. In this study, we generate high quality, virus-specific antisera by prime-boost immunization of 124 captive ERBs with Marburg virus, Ravn virus, Ebola virus, Bundibugyo virus, Taï Forest virus, Sudan virus or Reston virus. These virus-specific antisera, along with a set of 19 filovirus-naïve bat sera, are then used to validate a series of indirect ELISAs that utilize non-recombinant, infectious-based filovirus antigens for the detection of virus-specific IgG antibodies from bats. We then assess the level of serological cross-reactivity between each virus-specific antiserum and viral antigen and use this information to generate a filovirus antibody fingerprint that is able to predict which of the six filovirus species in our system is most antigenically similar to the species responsible for past infection.

## Methods

### Filovirus-specific antisera generated from ERBs

The bats used to generate the filovirus-specific antisera in this study originated from an ERB breeding colony^35^. A total of 124 ERBs were divided into seven groups (Table 1) and subcutaneously inoculated in the caudal abdominal region under isoflurane anesthesia with 4 log_10_TCID_50_ of Marburg virus, Ravn virus, Ebola virus, Taï Forest virus, Bundibugyo virus, Sudan virus or Reston virus (Table 2), prepared in 0.25 mL of sterile Dulbecco’s modified Eagle’s medium (Thermo Fisher Scientific Inc., Waltham, MA, USA). After eight weeks, the bat groups were subcutaneously challenged with 4 log_10_TCID_50_ of homologous virus. The bats were euthanized two to four weeks thereafter by cardiac exsanguination under isoflurane anesthesia, followed by an overdose of isoflurane. Whole blood collected into serum separator tubes (Fisher Scientific, Grand Island, NY, USA) at euthanasia was allowed to clot and then centrifuged at 1,500 g for 10 min. The sera were transferred into polypropylene CryoElite Wheaton vials (DWK Life Sciences, Millville, NJ, USA), and then temporarily stored under liquid nitrogen vapors in the biosafety level four laboratory (BSL-4) until they were double bagged, passed through a dunk tank containing 5% Micro-Chem Plus (National Chemical Laboratories Incorporated, Inc., Philadelphia, PA, USA) and transported to the gamma-cell irradiator. The doublebagged serum aliquots were sandwiched between layers of dry ice and then exposed to 5 × 10^6^ rads of gamma-cell irradiation using a cobalt-60 source.

**Table 1:** Descriptive analysis of the ERBs used in this study.

Group	Sex (%)			Age (%)	
	Male	Female	<1 yr	≥1 yr–≤5 yrs	>5 yrs
Marburg (n = 20)	0 (0.0)	20 (100.0)	0 (0.0)	13 (65.0)	7 (35.0)
Ravn (n = 20)	10 (50.0)	10 (50.0)	0 (0.0)	7 (35.0)	13 (65.0)
Ebola (n = 20)	0 (0.0)	20 (100.0)	0 (0.0)	15 (75.0)	5 (25.0)
Bundibugyo (n = 15)	4 (26.7)	11 (73.3)	15 (100.0)	0 (0.0)	0 (0.0)
Taï Forest (n = 15)	7 (46.7)	8 (53.3)	15 (100.0)	0 (0.0)	0 (0.0)
Sudan (n = 19)	4 (21.1)	15 (78.9)	0 (0.0)	6 (31.6)	13 (68.4)
Reston (n = 15)	15 (100.0)	0 (0.0)	2 (13.3)	9 (60.0)	4 (26.7)

**Table 2:** Details of the filovirus isolates used to inoculate the bats.

Filovirus	Isolate	Origin	Location	Year	Passage history
Marburg	200704852 Uganda Bat	*Rousettus aegyptiacus*	Uganda	2007	Vero E6 + 2
Ravn	200704669 Uganda Bat	*Rousettus aegyptiacus*	Uganda	2007	Vero E6 + 2
Ebola	Mayinga	Human	DRC	1976	Vero E6 + 2
Bundibugyo	200706291 Uganda prot	Human	Uganda	2007	Vero E6 + 3
Taï Forest	Cote d’Ivoire	Human	Cote d’Ivoire	1994	Vero E6 + 4, Huh7 + 1
Sudan	200011676 Gulu	Human	Uganda	2000–2001	Vero E6 + 3
Reston	H-28 Monkey R1036	Monkey	USA	1989	MA104 + 2, Vero E6 + 3, MA104 + 1, VC7 + 1, Vero E6 + 1

### Filovirus-naïve sera collected from ERBs

Sera obtained from filovirus-naïve bats from the breeding colony and negative control bats from previous experimental studies^33,35–37^ were used as filovirus-naïve sera. These sera were collected and processed in the same manner as outlined above.

### Ethics

All animal procedures were approved by the Institutional Animal Care and Use Committee (IACUC) and performed in compliance with the Guide for Laboratory Animal Care and Use (Committee for the Care and Use of Laboratory Animals 2011). The animal research facilities at the CDC have received full accreditation by Association for Assessment and Accreditation of Laboratory Animal Care (AAALAC).

### Biosafety

Procedures conducted with filoviruses or filovirus-infected bats were performed in accordance with Select Agent Regulations (Animal and Plant Health Inspection Service and Centers for Disease Control 2014) in a BSL-4 laboratory at the CDC. All bat cages were further contained within bio-flow isolator units with HEPA-filtered inlet and exhaust air supplies (Duo-Flow Mobile Units, Lab Products Inc., Seaford, DE, USA).

### Husbandry

All bats were group-housed according to filovirus inoculum in a climate controlled BSL-4 animal area, with a 12 h day/12 h night cycle. Bats received a quantity of fresh fruit, supplemented with protein/vitamin powder, equal to their body mass daily and water *ad libitum*.

### Virus and control antigen lysate preparation

Filovirus antigen lysates (Table 3) and uninfected control antigen lysate for the filovirus IgG indirect ELISAs were prepared as previously described^38–40^. Briefly, roller bottles of Vero E6 cells were inoculated with virus and the cultures were harvested when ≥90% of cells exhibited evidence of infection by immunofluorescence assay. Filovirus antigen lysates were prepared from the cultures by detergent basic buffer extraction of infected cells. Uninfected control antigen lysate was generated in the absence of virus, but otherwise prepared in the exact same manner.

**Table 3:** Details of the filovirus antigen lysates used in the indirect ELISAs.

Filovirus antigen lysate	Isolate	Origin	Location	Year
Marburg	200704852 Uganda Bat	*Rousettus aegyptiacus*	Uganda	2007
Ravn	Ravn-like	*Rousettus aegyptiacus*	Uganda	2007
Ebola	Zaire 9510621	Human	DRC	1995
Bundibugyo	200706291 Uganda prototype	Human	Uganda	2007
Taï Forest	Cote d’Ivoire 11/27/94	Human	Cote d’Ivoire	1994
Sudan	Gulu	Human	Uganda	2000–2001
Reston	200900831 Philippine prototype	Pig	Philippines	2009

### Filovirus IgG indirect ELISAs

Each filovirus-specific antiserum (n = 124) and filovirus-naïve serum (n = 19) was tested for reactivity against each filovirus antigen lysate (n = 7) and uninfected control antigen lysate (n = 2; Supplementary Fig. S1). Wells of 96-well ELISA plates were coated (100 µL) with the dilution of filovirus antigen lysate (diluent: PBS containing 1% thimerosal) that was found to result in optimal reactivity with sera pooled from each of the filovirus-infected bat groups (1:1000 for the Ebola, Bundibugyo, Taï Forest and Reston virus lysates, and 1:2000 for the Marburg, Ravn and Sudan virus lysates) and corresponding wells were coated with an equivalent dilution of uninfected control lysate (1:1000 or 1:2000). After incubation overnight at 4 °C, the plates were washed with PBS containing 0.1% Tween-20 (PBS-T) and 100 µL of serum diluent (PBS containing 5% skim milk and 0.1% tween-20) was added to each well of the plate. After 10 min, 33 µL of a 21:521 dilution of gamma-irradiated bat serum pre-diluted in masterplate diluent (PBS containing 5% skim milk powder, 0.5% tween-20 and 1% thimerosal) was added to the first well of the plate and four-fold serial dilutions were performed. Final bat serum concentrations were 1:100, 1:400, 1:1600 and 1:6400. Following a 1 hr incubation at 37 °C, the plates were washed with PBS-T and 100 µL of a 1:20,000 dilution of goat anti-bat IgG conjugated to horseradish peroxidase (Bethyl Laboratories, Montgomery, TX, USA, Cat#: A140-118P, Lot#: A140-118P-13) in serum diluent was added to the plates. The manufacturer notes that this antibody reacts specifically with bat IgG and with light chains common to other immunoglobulins. After incubation for 1 hr at 37 °C, the plates were washed with PBS-T, 100 µL of the Two-Component ABTS Peroxidase System (KPL, Gaithersburg, MD, USA) was added, and the plates were allowed to incubate for 30 min at 37 °C. The plates were then read on a microplate spectrophotometer set at 410 nm. The optical density (OD) values of each four-fold serial dilution were visually inspected to ensure linearity. To negate non-specific background reactivity, adjusted OD values were calculated by subtracting the ODs at each four-fold dilution of wells coated with uninfected control antigen lysate from ODs at corresponding wells coated with filovirus antigen lysate. The adjusted sum OD value was determined by summing the adjusted OD values at each four-fold serial dilution. The average adjusted sum OD of duplicate runs was reported. Notably, performing each indirect ELISA (seven filovirus antigen lysates and two dilutions of uninfected control antigen lysate) in duplicate required only 25 µL of serum.

### Influence of sex and age on the reactivity of antisera with homologous antigen

The breeding colony was founded from Ugandan wild-caught, filovirus-naïve adult (≥1 yr) ERBs^35^. For founder bats, age in years was determined by subtracting the date of capture from the date of euthanasia and then adding one year. For bats born in captivity, age in years was determined by subtracting the date of birth from the date of euthanasia. The bats were then classified into three age categories: <1 yr, ≥1 yr–≤5 yrs and >5 yrs. A two-way ANOVA was performed to determine if serological reactivity of bat sera with homologous antigen was significantly influenced (p < 0.05) by sex, age category, or the interaction between sex and age category (SPSS Statistics 21, IBM Software, Armonk, NY, USA).

### Determination of seropositivity cutoff values

For each filovirus-specific IgG indirect ELISA, quadratic discriminant analysis was used to determine a cutoff value for seropositivity by considering: (1) the reactivity of the filovirus-specific antisera group with homologous antigen, (2) the reactivity of the filovirus-naïve sera group with that same antigen, and (3) the prior probability of the filovirus-specific antisera group (VBA for Microsoft Access 2016, Microsoft Office Professional Plus 2016, Redmond, WA, USA). The prior probability of each filovirus-specific antisera group was set at 0.15. This value represents the overall population seroprevalence of marburgvirus in its natural reservoir host, the ERB^18^. We assume that the seroprevalence of the ebolaviruses in their respective natural reservoir bat hosts approximates this value. However, the prior probability has relatively little influence on determining the magnitude of the cutoff value for seropositivity. The cutoff value for each filovirus-specific indirect ELISA represents the value where the posterior probabilities for the reactivity of the filovirus-specific antisera with homologous antigen and the reactivity of filovirus-naïve sera with this same antigen are equal.

### Sensitivity and specificity of the indirect ELISAs

For each indirect ELISA at its specified seropositivity cutoff value, the filovirus-specific antiserum and filovirus-naïve serum samples were classified as true positive, false positive, false negative or true negative. The sensitivity of each assay was then calculated by dividing the number of individuals with a positive test result by the number of individuals with a history of past infection (experimentally inoculated with that particular virus), and the specificity of each assay was calculated by dividing the number of individuals without a history of past infection (filovirus-naïve group) by the number of individuals with a negative test result.

### Level of serological cross-reactivity

The level of serological cross-reactivity between each group of filovirus-specific antisera and each heterologous virus antigen was calculated by dividing the number of antisera positive for reactivity with a virus antigen by the total number of antisera tested against that antigen (e.g., number of Marburg virus antisera positive for reactivity with Ravn virus antigen/number of Marburg virus antisera tested for reactivity against Ravn antigen), and was expressed as a percentage.

### Relationship between serological cross-reactivity and virus protein amino acid identity

After the nucleotide sequences of the filovirus isolates used to inoculate the bats in this study were retrieved from GenBank, the coding regions were translated into amino acids and aligned using the MUSCLE algorithm (Geneious 9.1.2, Biomatters Limited, Auckland, New Zealand). Percent identity values were then calculated from pairwise amino acid comparisons generated from the virus protein alignment. A Pearson’s Product-Moment Correlation was performed to assess the relationship between percent serological cross-reactivity and percent amino acid identity (SPSS Statistics 21, IBM Software, Armonk, NY, USA).

### Antibody fingerprint analysis

Quadratic discriminant analysis was used to predict which one of the seven filoviruses in the system was most antigenically similar to the filovirus responsible for past infection by considering the relative reactivity of each filovirus-specific antiserum and filovirus-naïve serum with each filovirus antigen, as well as their covariance (Visual Basic for Microsoft Access). First, prior probabilities were calculated for the eight classes used in the analysis (seven filovirus antigen classes and one negative class). Second, classification was performed by calculating posterior probabilities for the inclusion of an antisera/sera sample in each class and then assigning the sample to the class with the highest probability.

### Evaluating the performance and discriminatory ability of the filovirus IgG indirect ELISA system

To evaluate the ability of our system comprising seven filovirus-specific indirect ELISAs to predict the filovirus species most antigenically similar to the species responsible for past infection, we tested seven Marburg virus convalescent serum^35^ or whole blood^36^ samples collected from experimentally inoculated ERBs. Five of these samples were collected four weeks post primary Marburg virus inoculation^35,36^, while two of the samples were collected at 23 and 27 weeks post primary inoculation following a “natural” boost (i.e., Marburg virus-specific antibody levels waned in these bats and then increased following contact with infectious cagemates)^36^.

## Results

### Serological reactivity with homologous filovirus antigen is not influenced by age or sex

A total of 124 ERBs were used in this study that was performed over a two-year time span. The total number of bats dedicated to this study, as well as the size and composition of each group was dependent on the reproductive capacity of the ERB breeding colony, as well as the characteristics and number of bats needed for other experimental studies. The 124 bats were divided into seven groups, with group sizes ranging from 15–20 individuals (Table 1). Two groups of bats had approximately equal sex ratios (Ravn and Taï Forest), while two groups were comprised of mostly female individuals (Bundibugyo and Sudan), and the remaining five groups were comprised of all male (Reston) or all female (Marburg and Ebola) individuals. Two groups of bats were comprised solely of individuals <1 yr of age (Bundibugyo and Taï Forest), while the majority of individuals in three groups were ≥1- ≤5 yrs of age (Marburg, Ebola and Reston) and the majority of individuals in two groups were >5 yrs of age (Ravn and Sudan). Despite the observed variability in group composition, a two-way ANOVA revealed that serological reactivity with homologous filovirus antigen was not significantly (p > 0.05) influenced by sex (F(1,118) = 1.293), age (F(2,118) = 0.837), or the interaction between sex and age (F(2,118) = 0.715).

### Filovirus-specific indirect ELISAs are highly sensitive and specific

**Figure 1:**
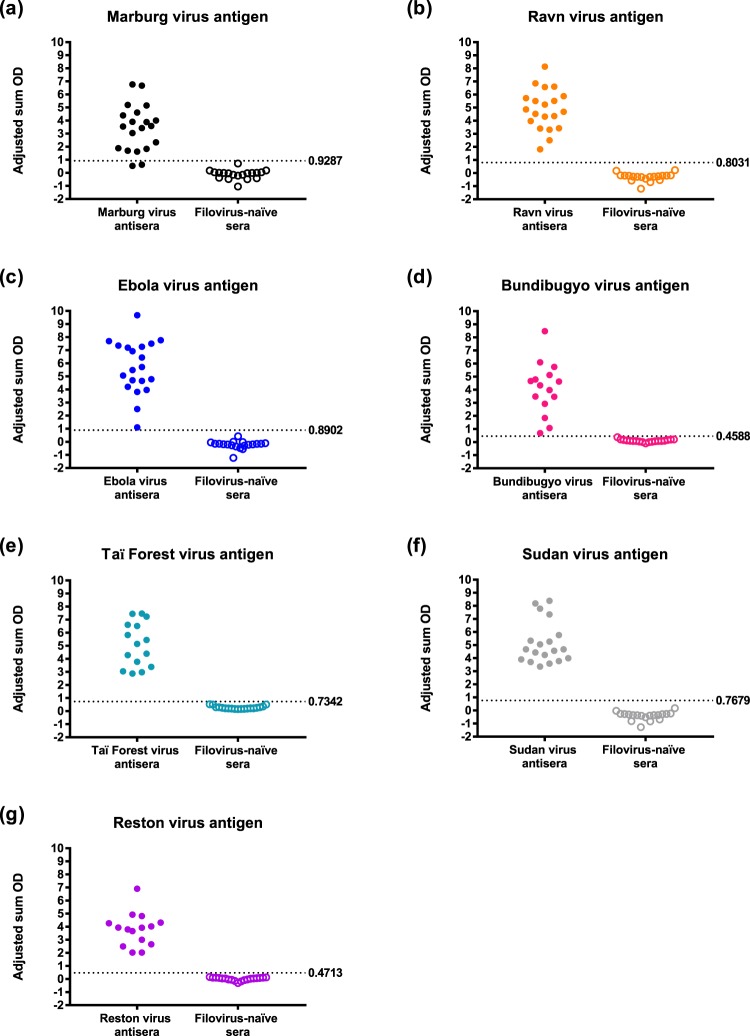
IgG serological reactivity of filovirus-specific bat antisera and filovirus-naïve bat sera with homologous filovirus antigens. Panels represent reactivity of filovirus-specific bat antisera and filovirus-naïve bat sera with: (**a**) Marburg virus antigen, (**b**) Ravn virus antigen, (**c**) Ebola virus antigen, (**d**) Bundibugyo virus antigen, (**e**) Taï Forest virus antigen, (**f**) Sudan virus antigen and (**g**) Reston virus antigen. Closed circles represent the adjusted sum OD value for an individual bat. The dashed lines and numbers to the right of the lines represent the cutoff values of the assays. An individual was classified as seropositive if their adjusted sum OD value was ≥ the cutoff value of that particular assay. Refer to Table 4 for statistics on the reactivity of each filovirus antigen lysate with homologous antisera and filovirus-naïve sera, as well as for the sensitivity and specificity of each of the filovirus IgG indirect ELISA.

With the exception of the IgG antibody indirect ELISA using Marburg virus antigen (90% sensitivity and 100% specificity; Fig. 1a and Table 4), all of the assays exhibited 100% sensitivity and specificity (Fig. 1 and Table 4). The mean group adjusted sum OD values ranged from 3.43 (±1.77 SD) for the IgG antibody indirect ELISA using Marburg virus antigen to 5.69 (±2.06 SD) for the IgG antibody indirect ELISA using Ebola virus antigen.

**Table 4:** Sensitivity and specificity of the filovirus IgG indirect ELISAs.

Filovirus antigen lysate	Cutoff	Reactivity with homologous antisera		Reactivity with filovirus-naïve sera		Sensitivity (%)	Specificity (%)
		Mean group adjusted sum OD (SD)	Proportion reactive	Mean group adjusted sum OD (SD)	Proportion reactive		
Marburg	0.9287	3.43 (1.77)	18/20	−0.12 (0.36)	0/19	90	100
Ravn	0.8031	4.86 (1.56)	20/20	−0.31 (0.31)	0/19	100	100
Ebola	0.8902	5.69 (2.06)	20/20	−0.23 (0.32)	0/19	100	100
Bundibugyo	0.4588	4.08 (2.00)	15/15	0.10 (0.10)	0/19	100	100
Taï Forest	0.7342	5.09 (1.69)	15/15	0.27 (0.12)	0/19	100	100
Sudan	0.7679	5.16 (1.61)	15/15	−0.42 (0.32)	0/19	100	100
Reston	0.4713	3.78 (1.27)	15/15	0.01 (0.12)	0/19	100	100

### Strong positive correlation between filovirus serological cross-reactivity and filovirus protein amino acid identity

**Figure 2:**
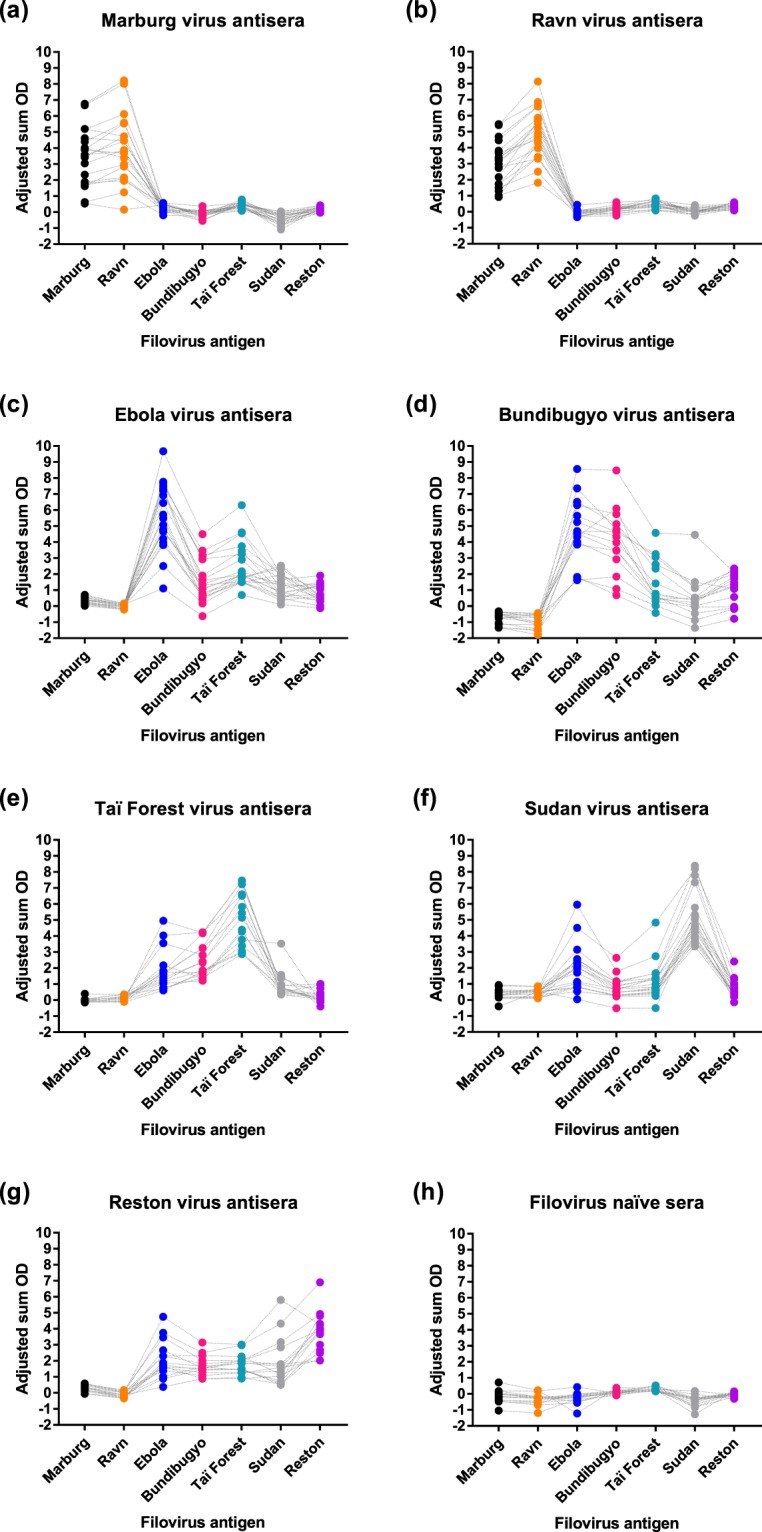
IgG serological cross-reactivity of filovirus-specific bat antisera with heterologous filovirus antigens. Panels represent reactivity of filovirus antigens with: (**a**) Marburg virus antisera, (**b**) Ravn virus antisera, (**c**) Ebola virus antisera, (**d**) Bundibugyo virus antisera, (**e**) Taï Forest virus antisera, (**f**) Sudan virus antisera and (**g**) Reston virus antisera. Closed circles represent the adjusted sum OD value for an individual bat antiserum against each of the heterologous filovirus antigens. Dotted lines are available to track the reactivity of an individual bat antiserum against the six heterologous filovirus antigens. Refer to Table 5 for statistics on the cross-reactivity between each filovirus-specific bat antisera and heterologous filovirus antigen.

Figure 2 and Table 5 show the level of serological cross-reactivity between each group of bat filovirus-specific antisera and the six heterologous filovirus antigens. The consistent magnitudes of serological reactivity of the individual bat antisera across all six heterologous filovirus antigens highlights the robust performance of our filovirus IgG indirect ELISA system. Strong serological cross-reactivity was observed between Marburg virus antisera and Ravn virus antigen (95%), and Ravn virus antisera and Marburg virus antigen (95%) (Fig. 2a,b). The level of serological cross-reactivity between ebolavirus antisera and ebolavirus antigen varied from 20% (Taï Forest virus antisera versus Reston virus antigen) to 100% (Bundibugyo virus antisera versus Ebola virus antigen, Taï Forest virus antisera versus Bundibugyo virus antigen, and Reston virus antisera versus Bundibugyo and Taï Forest virus antigens) (Fig. 2e–g). Very limited cross-reactivity was observed between marburgvirus antisera and ebolavirus antigen (0%: majority of antisera-antigen combinations; 5%: Marburg virus antisera versus Taï Forest virus antigen, Ravn virus antisera versus Bundibugyo and Taï Forest virus antigens; 15%: Ravn virus antisera versus Reston virus antigen), and ebolavirus antisera tested against marburgvirus antigen (5.3%: Sudan virus antisera versus Marburg virus antigen; 10.3% Sudan virus antisera versus Ravn virus antigen) (Fig. 2). A Pearson’s Product-Moment Correlation revealed that there was a statistically significant, strong positive correlation between percent filovirus serological cross-reactivity and percent filovirus amino acid identity (r = 0.922, n = 42, p < 0.01).

**Table 5:** Level of IgG serological reactivity between virus-specific bat antisera and heterologous filovirus antigen.

Virus-specific antisera	Virus antigen lysate	Proportion cross-reactive	% Cross-reactive
Marburg (n = 20)	Ravn	19/20	95.0
	Ebola	0/20	0.0
	Bundibugyo	0/20	0.0
	Taï Forest	1/20	5.0
	Sudan	0/20	0.0
	Reston	0/20	0.0
Ravn (n = 20)	Marburg	19/20	95.0
	Ebola	0/20	0.0
	Bundibugyo	1/20	5.0
	Taï Forest	1/20	5.0
	Sudan	0/20	0.0
	Reston	3/20	15.0
Ebola (n = 20)	Marburg	0/20	0.0
	Ravn	0/20	0.0
	Bundibugyo	18/20	90.0
	Taï Forest	19/20	95.0
	Sudan	14/20	70.0
	Reston	14/20	70.0
Bundibgyo (n = 15)	Marburg	0/15	0.0
	Ravn	0/15	0.0
	Ebola	15/15	100.0
	Taï Forest	8/15	53.3
	Sudan	4/15	26.7
	Reston	12/15	80.0
Taï Forest (n = 15)	Marburg	0/15	0.0
	Ravn	0/15	0.0
	Ebola	12/15	80.0
	Bundibugyo	15/15	100.0
	Sudan	6/15	40.0
	Reston	3/15	20.0
Sudan (n = 19)	Marburg	1/19	5.3
	Ravn	2/19	10.5
	Ebola	13/19	68.4
	Bundibugyo	13/19	68.4
	Taï Forest	12/19	63.2
	Reston	11/19	57.9
Reston (n = 15)	Marburg	0/15	0.0
	Ravn	0/15	0.0
	Ebola	13/15	86.7
	Bundibugyo	15/15	100.0
	Taï Forest	15/15	100.0
	Sudan	11/15	73.3

### Filovirus serological cross-reactivity dataset used to develop an antibody fingerprint classification system

We used the adjusted sum OD data (each serum tested against each antigen) generated from bats intentionally infected with each of the seven known culturable filoviruses to develop a classification system (fingerprint) to predict which filovirus elicited the antibody response. Supplementary Table S1 shows posterior probability support values for classification of each filovirus-specific antiserum or filovirus-naïve serum sample into eight pre-defined classes (seven filovirus antigen classes and one negative class), with probabilities of each row in the table summing to 1.00000. Each sample was assigned to the class with the highest posterior probability (i.e., highest degree of certainty). Overall, all of the samples (143/143) were correctly classified, with posterior probabilities in support of the true class (i.e., filovirus used to prime-boost each bat) ranging from 0.97636 to 1.00000. This indicates that this system, using seven independent filovirus IgG indirect ELISAs, has the ability to establish an antibody fingerprint from filovirus convalescent bat sera that can be used to predict which of the filovirus species in the system is the most antigenically similar to the species responsible for past infection (see Supplementary Note for an example of how the antibody fingerprinting was performed in this study).

### Identification of the filovirus species most likely responsible for past infection

**Figure 3:**
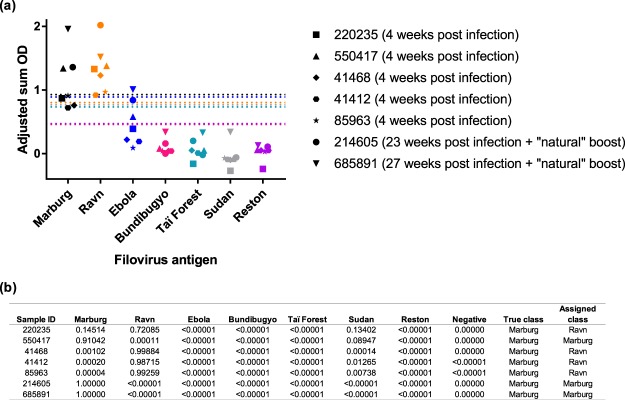
Performance and discriminatory ability of the filovirus IgG indirect ELISA system. (**a**) IgG serological cross-reactivity of Marburg virus-specific bat antisera collected after primary experimental inoculation with Marburg virus or following primary experimental inoculation with Marburg virus and a “natural” boost from infectious cagemates. Distinct symbol shapes represent the adjusted sum OD value for an individual bat antiserum against each of the filovirus antigens. The dashed lines are colored according to filovirus antigen and represent the cutoff value for each of the assays. (**b**) Posterior probability support values for classification of each of the samples represented in the above panel into each antigen class.

To further assess the performance and predictive ability of our filovirus IgG indirect ELISA system, we tested five Marburg virus convalescent sera or whole blood samples collected four weeks post primary Marburg virus experimental inoculation and two whole blood samples collected at 23 and 27 weeks post primary experimental inoculation following a “natural” boost (i.e., Marburg virus-specific antibody levels waned and then increased following contact with infectious cagemates). As expected, marburgvirus-specific IgG antibody levels were markedly lower in these seven convalescent serum and whole blood samples compared to marburgvirus-specific IgG antibody levels in the serum samples from the prime-boosted bats in this study (Fig. 3a). However, all samples collected after primary Marburg virus inoculation or following a “natural” boost were classified as marburgvirus IgG antibody positive. The antibody fingerprint analysis predicted that three of these bats were previously infected with Marburg virus (Sample IDs 550417, 214605 and 685891 with Marburg posterior probability values of 0.91042, 1.00000, and 1.00000, respectively) and four were previously infected with Ravn virus (Sample IDs 220235, 41468, 41412 and 85963 with Ravn posterior probability values of 0.72085, 0.99884, 0.98715 and 0.99259, respectively; Fig. 3b).

## Discussion

In this study, we generated filovirus-specific antisera from prime-boosted ERBs and collected filovirus-naïve sera to validate and characterize an indirect ELISA system that utilizes non-recombinant, infectious-based filovirus antigens for the detection of virus-specific IgG antibodies from bats. Initial validation of the system revealed that the individual filovirus-specific IgG indirect ELISAs exhibited high sensitivity (90–100%) and specificity (100%). Similar to previous studies using human sera, we observed limited to no serological IgG cross-reactivity between the filovirus genera (0–15%)^41–43^, varying levels of serological IgG cross-reactivity between the ebolavirus species (20–100%)^40,44^ and strong serological IgG cross-reactivity within the species *Marburg marburgvirus* (95%). The low level of serological IgG cross-reactivity between some of the ebolavirus species underscores the necessity of performing all assays within the system to avoid false negative results. Although significant levels of serological IgG cross-reactivity were observed between the prime-boost filovirus-specific antisera and some of the filovirus antigens, when the overall covariance of the seven-individual indirect ELISAs in the system were considered, we were able to predict the filovirus species responsible for past infection 100% of the time using as little as 25 μL of sera (each serum was tested against each antigen in duplicate). Further evaluation of the performance and predictive ability of our system using seven Marburg virus convalescent serum or whole blood samples collected after primary experimental infection with Marburg virus (or primary experimental infection with Marburg virus plus a “natural” boost) confirmed that the system was able to predict the filovirus species most likely responsible for past infection. As expected, the filovirus IgG indirect ELISA system was not able to correctly predict whether past infection was due to Marburg virus or Ravn virus 100% of the time. This finding supports the current classification of Marburg and Ravn viruses into a single virus species, *Marburg marburgvirus*^2^.

The robustness and discriminatory power of our system results from its underlying components and various control points. We employed infectious-based filovirus antigens, rather than recombinant virus protein antigens, as a strategy to increase both the sensitivity (i.e., ability of the filovirus IgG indirect ELISA system to correctly identify those with past exposure to a filovirus as filovirus IgG antibody positive) and specificity (i.e., ability of the filovirus IgG indirect ELISA system to correctly identify those with no past exposure to a filovirus as filovirus IgG antibody negative) of the system. Although recombinant filovirus antigens can be generated in large quantities and do not require a BSL-4 laboratory for production, the use of single recombinant virus proteins for antibody detection can lead to false negative results if an individual’s antibody repertoire is not directed against the particular recombinant virus protein that was employed^23,26–31^. Alternatively, false positive serological results can occur when recombinant virus antigens unknowingly share similar epitopes with other virus antigens for which the study population possesses antibodies against^45,46^. False positive results can also arise from failing to account for non-specific serological reactivity resulting from the presence of non-virus contaminants in an antigen preparation. Here, we negated non-specific serological reactivity to Vero E6 cell components by subtracting the reactivity of wells coated with uninfected antigen lysate from the reactivity of corresponding wells coated with filovirus antigen lysate. While the majority of previously published ebolavirus serosurveys of bats used recombinant filovirus antigens generated in bacterial expression systems, not all of the studies implemented procedures to negate non-specific reactivity between residual bacterial epitopes in the antigen preparation and antibacterial antibodies in bat sera^27,29^. Like the majority of published ebolavirus serosurveys of bats, we performed serial serum dilutions to assess for linearity and specificity^22,24,25,27–30^. Most importantly, we included pooled filovirus-specific bat antisera as a positive control and pooled filovirus-naïve bat sera as a negative control in every run to ensure that our filovirus IgG indirect ELISA system continually performed as expected.

Using filovirus-specific antisera collected from bats that were prime-boosted at the same time not only allowed us to thoroughly examine the level of serological cross-reactivity between the virus-specific antisera and heterologous filovirus antigen, but also provided the opportunity to use this knowledge to evaluate previously published ebolavirus serosurveys of bats^22–32^. Four out of the 11 previously published ebolavirus serosurveys of bats used Ebola virus antigen only to detect ebolavirus IgG antibodies^22,24–26^ and another four of these serosurveys used Ebola and Reston antigens only to detect ebolavirus IgG antibodies^23,27–29^. Based on the low serological cross-reactivity between Ebola virus antigen and Sudan virus antisera (68.4%) and Reston virus antigen and Sudan virus antisera (57.7%) observed in our study, we predict that the serosurveys using Ebola virus antigen or Ebola and Reston virus antigens only would have missed the opportunity to detect bats previously infected with Sudan virus. Likewise, due to the high level of serological cross reactivity between ebolavirus-specific antisera and heterologous ebolavirus antigen that we observed in our study, it is possible that the serological reactivity of bat sera with Ebola or Reston virus antigens in the previous serosurveys was due to past infection with one of the other known ebolaviruses or an undescribed ebolavirus. Although we successfully used quadratic discriminant analysis to predict which of the filovirus species in the system was most antigenically similar to the species responsible for past infection, only nucleotide sequence data can be used to ascertain infection with a particular filovirus. Furthermore, incrimination of an ebolavirus-natural reservoir host relationship requires consistent detection of both active (RNA and virus isolation from tissues/bodily fluids suggestive of a transmission mechanism) and past infection with a particular ebolavirus through space and time.

While our filovirus IgG indirect ELISA system enables the robust detection of virus-specific IgG antibodies from bats and was able to predict the filovirus species most likely responsible for past infection, it does have some limitations. First, this system was validated using virus-specific antisera from prime-boosted bats and sera from filovirus-naïve bats. Although all seven of the Marburg virus convalescent whole blood and serum samples collected from ERBs after primary experimental Marburg virus inoculation or following a “natural” boost were marburgvirus IgG antibody positive, these samples had markedly lower marburgvirus-specific IgG antibody levels compared to samples collected from the bats in this study that were prime-boosted with one of seven filoviruses. This suggests that the sensitivity of the individual filovirus IgG indirect ELISAs may be lower than what was reported here (90–100%) using filovirus-specific antisera from prime-boosted bats and filovirus naïve bat sera. However, statistical determination of seropositivity cutoff values for the indirect ELISAs were largely driven by the variance surrounding the mean reactivity of the filovirus-naïve sera with each of the virus antigens. It is likely that circulation and long-term maintenance of the ebolaviruses in their natural reservoir hosts leads to multiple virus exposures throughout the lifetime of an individual, resulting in boosting of virus-specific IgG antibody levels. “Natural” boosting of virus-specific IgG antibody levels in ERBs that had been previously infected with Marburg virus was observed during a recent experimental transmission study shortly after documenting virus infection and seroconversion in naïve contact bats^36^. Others have reported periodic boosting of rabies virus neutralizing antibody titers in a wild colony of big brown bats (*Eptesicus fuscus*)^47^. Second, we used a goat anti-bat IgG conjugated to horseradish peroxidase as the secondary antibody in our system. This antibody was generated using sera from ERBs, as well as sera from nine other bat species comprising four chiropteran families (manufacturer product datasheet). While this antibody has been confirmed to react with sera from these 10 chiropteran species and has been used with success to detect IgG antibodies in sera from >27 bat species comprising seven chiropteran families^48–57^, it is possible that its reactivity with IgG antibodies from divergent bat species might diminish. If this is the case, the concentration of secondary antibody may need to be optimized prior to testing a new species of bat or an alternative secondary antibody may need to be used. Third, our filovirus IgG indirect ELISA system includes seven virus antigens that represent all of the reported culturable filoviruses. Given the recent discoveries of filovirus RNA from divergent bat families in geographically distant locations (Lloviu virus from *Miniopterus schreibersii* [family Miniopteridae] in Spain^58^ and Hungary^59^; Bombali virus from *Chaerephon pumilus* [family Molossidae] and *Mops condylurus* [family Molossidae] in Sierra Leone^4^; Měnglà virus from a *Rousettus* bat [family Pteropodidae] in China^60^), we expect that there are a number of undiscovered ebolaviruses that continue to circulate in nature. However, we believe that the genetic diversity of ebolavirus antigens (up to 61.6%) included in our system will allow for the detection of past infection with a novel ebolavirus, and prediction of the ebolavirus species that is most antigenically similar to the species responsible for past infection.

We intend to use our filovirus IgG indirect ELISA system to screen archived and incoming specimens from cross-sectional serosurveys of the bat population of sub-Saharan Africa and Asia for evidence of past filovirus infection. If other specimen types exist for bat species identified as filovirus seropositive, they will be tested for evidence of active infection using virus-specific qRT-PCR, pan-filovirus RT-PCR and/or virus isolation techniques. Bat species positive for evidence of active filovirus infection or species exhibiting a seroprevalence equivalent to or larger than that of marburgvirus in ERBs (~15% total population seroprevalence) will be targeted for longitudinal studies aimed at collecting a wide range of specimens for evidence of active and past ebolavirus infection.

While most of the virus-specific antisera generated in this study will be pooled to use as positive controls in our indirect filovirus ELISA system, some will be reserved for investigations aimed at determining the mechanisms by which bats clear and control filovirus infections through virus neutralization and cross-neutralization assays, as well as epitope mapping studies. Any remaining filovirus-specific bat antisera may be used to develop a pan-ebolavirus indirect ELISA that utilizes a mixture of non-recombinant, infectious based antigens from culturable ebolaviruses, as well as validate the performance of indirect ELISAs that use recombinant filovirus proteins to detect filovirus antibodies.

## Supplementary information

Supplementary information

## Data Availability

The authors declare that all data supporting the findings of this study are available within the article or from the corresponding author upon request.
