# Supplementary material for: Comparative analysis of serologic cross-reactivity using convalescent sera from filovirus-experimentally infected fruit bats
Source: Sci Rep. 2019 Apr 30;9:6707. doi: 10.1038/s41598-019-43156-z (PMC6491471; doi:10.1038/s41598-019-43156-z)
Supplement: Supplementary file 1 — Supplementary information [file 41598_2019_43156_MOESM1_ESM.pdf]

Comparative analysis of serologic cross-reactivity using convalescent sera from filovirus-experimentally infected fruit bats

Amy J. Schuh[1,2], Brian R. Amman[1], Tara S. Sealy[1], Timothy D. Flietstra[1], Jonathan C. Guito[1], Stuart T. Nichol[1] and Jonathan S. Towner[1,3,*]

[1]Viral Special Pathogens Branch, Division of High-Consequence Pathogens and Pathology, Centers for Disease Control and Prevention, Atlanta, GA, 30329, USA
[2]Commissioned Corps, United States Public Health Service, Rockville, MD, 20852, USA
[3]Department of Pathology, College of Veterinary Medicine, University of Georgia, Athens, GA, 30602, USA

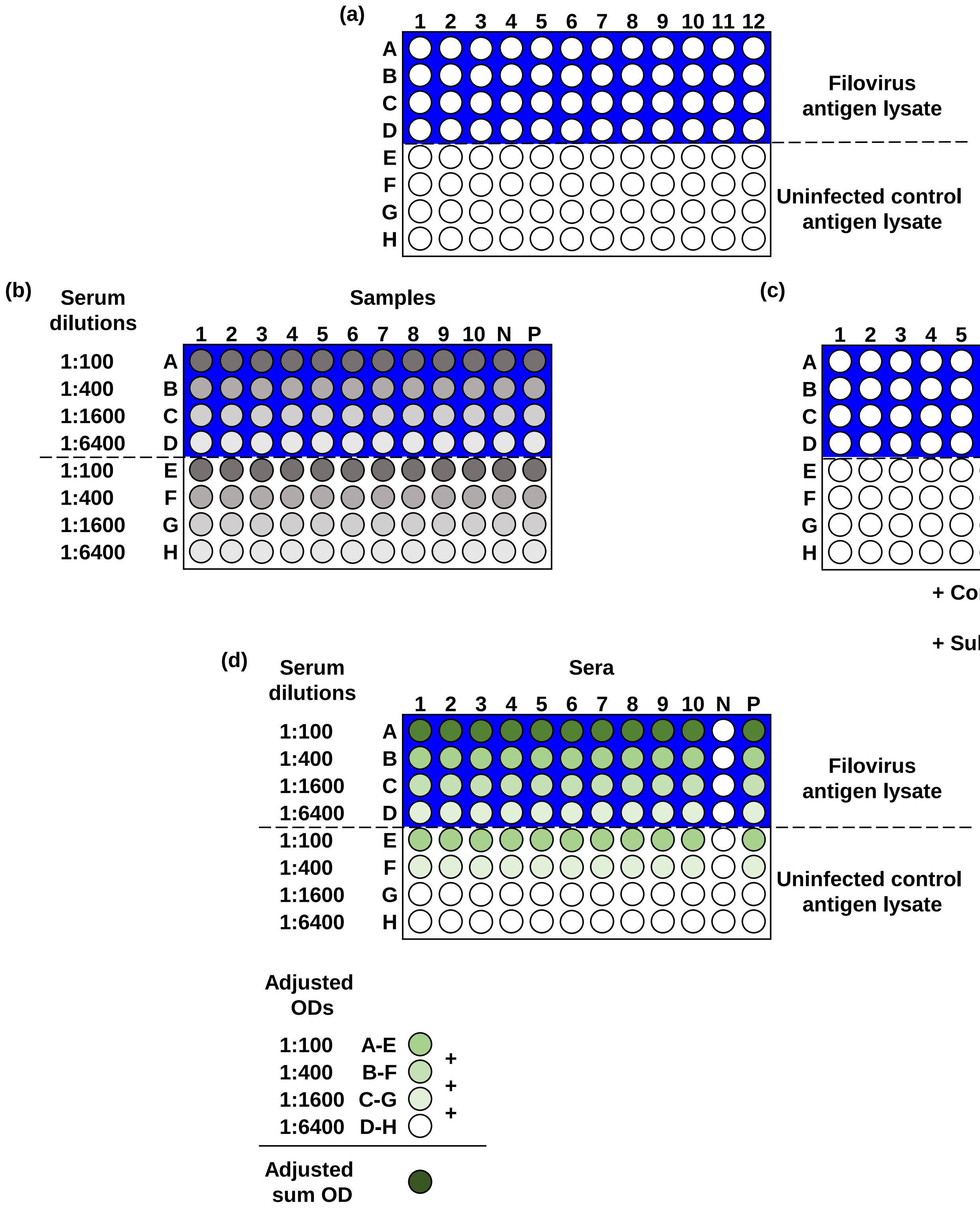

Supplementary Figure S1. Performance of filovirus IgG indirect ELISAs**.** (a) Coat wells in rows A-D of a 96-well plate with 100 µL filovirus antigen lysate, coat wells in rows E-F with 100 µL uninfected control lysate and then incubate the plate overnight at 4ºC. (b) Wash the plate and then add 100 µL of serum diluent to each well. Add 33 µL of bat serum, negative control (N) and positive control (P) samples pre-diluted in masterplate diluent (21:521 dilution) to wells in row A and then to corresponding wells in row E. Perform four-fold serial dilutions of the serum samples on both sides of the plate and then incubate the plate for 1 hr at 37ºC. (c) Wash the plate, add 100 µL of goat anti-bat IgG conjugated to horseradish peroxidase to each well and then incubate the plate for 1 hr at 37ºC. Wash the plate, add 100 µL of the Two-Component ABTS Peroxidase System to each well of the plate and then incubate the plate for 30 min at 37ºC. (d) Read the plate on a spectrophotometer. Calculate adjusted optical density (OD) values for each sample by subtracting the ODs at each four-fold dilution of wells coated with uninfected control antigen lysate from ODs at corresponding wells coated with filovirus antigen lysate. Calculate the adjusted sum OD for each sample by summing the adjusted OD values at each four-fold serial dilution.

Supplementary Table S1. Posterior probability support values for classification of each filovirus-specific bat antiserum sample into each filovirus antigen class.

Sample ID	Marburg	Ravn	Ebola	Bundibugyo	Taï Forest	Sudan	Reston	Negative	Assigned class	True class
215330	1.00000	<0.00001	<0.00001	<0.00001	<0.00001	<0.00001	<0.00001	<0.00001	Marburg	Marburg
219792	1.00000	<0.00001	<0.00001	<0.00001	<0.00001	<0.00001	<0.00001	<0.00001	Marburg	Marburg
438185	1.00000	<0.00001	<0.00001	<0.00001	<0.00001	<0.00001	<0.00001	<0.00001	Marburg	Marburg
438440	0.99988	<0.00001	0.00004	<0.00001	<0.00001	<0.00001	0.00007	<0.00001	Marburg	Marburg
440976	1.00000	<0.00001	<0.00001	<0.00001	<0.00001	<0.00001	<0.00001	<0.00001	Marburg	Marburg
441384	0.99996	0.00004	<0.00001	<0.00001	<0.00001	<0.00001	<0.00001	<0.00001	Marburg	Marburg
441951	0.99998	0.00002	<0.00001	<0.00001	<0.00001	<0.00001	<0.00001	<0.00001	Marburg	Marburg
442331	1.00000	<0.00001	<0.00001	<0.00001	<0.00001	<0.00001	<0.00001	<0.00001	Marburg	Marburg
443629	1.00000	<0.00001	<0.00001	<0.00001	<0.00001	<0.00001	<0.00001	<0.00001	Marburg	Marburg
546951	0.99999	0.00001	<0.00001	<0.00001	<0.00001	<0.00001	<0.00001	<0.00001	Marburg	Marburg
547158	1.00000	<0.00001	<0.00001	<0.00001	<0.00001	<0.00001	<0.00001	<0.00001	Marburg	Marburg
642904	0.99429	0.00571	<0.00001	<0.00001	<0.00001	<0.00001	<0.00001	<0.00001	Marburg	Marburg
656933	1.00000	<0.00001	<0.00001	<0.00001	<0.00001	<0.00001	<0.00001	<0.00001	Marburg	Marburg
684800	1.00000	<0.00001	<0.00001	<0.00001	<0.00001	<0.00001	<0.00001	<0.00001	Marburg	Marburg
685742	1.00000	<0.00001	<0.00001	<0.00001	<0.00001	<0.00001	<0.00001	<0.00001	Marburg	Marburg
691089	1.00000	<0.00001	<0.00001	<0.00001	<0.00001	<0.00001	<0.00001	<0.00001	Marburg	Marburg
691313	1.00000	<0.00001	<0.00001	<0.00001	<0.00001	<0.00001	<0.00001	<0.00001	Marburg	Marburg
725971	1.00000	<0.00001	<0.00001	<0.00001	<0.00001	<0.00001	<0.00001	<0.00001	Marburg	Marburg
726247	1.00000	<0.00001	<0.00001	<0.00001	<0.00001	<0.00001	<0.00001	<0.00001	Marburg	Marburg
726539	0.99991	0.00009	<0.00001	<0.00001	<0.00001	<0.00001	<0.00001	<0.00001	Marburg	Marburg
214766	<0.00001	1.00000	<0.00001	<0.00001	<0.00001	<0.00001	<0.00001	<0.00001	Ravn	Ravn
440718	<0.00001	1.00000	<0.00001	<0.00001	<0.00001	<0.00001	<0.00001	<0.00001	Ravn	Ravn
441239	<0.00001	1.00000	<0.00001	<0.00001	<0.00001	<0.00001	<0.00001	<0.00001	Ravn	Ravn
441257	0.00001	0.99999	<0.00001	<0.00001	<0.00001	<0.00001	<0.00001	<0.00001	Ravn	Ravn
441317	0.00005	0.99995	<0.00001	<0.00001	<0.00001	<0.00001	<0.00001	<0.00001	Ravn	Ravn
441344	<0.00001	1.00000	<0.00001	<0.00001	<0.00001	<0.00001	<0.00001	<0.00001	Ravn	Ravn
441363	<0.00001	1.00000	<0.00001	<0.00001	<0.00001	<0.00001	<0.00001	<0.00001	Ravn	Ravn
441447	<0.00001	1.00000	<0.00001	<0.00001	<0.00001	<0.00001	<0.00001	<0.00001	Ravn	Ravn
441974	<0.00001	1.00000	<0.00001	<0.00001	<0.00001	<0.00001	<0.00001	<0.00001	Ravn	Ravn
442338	0.00003	0.99997	<0.00001	<0.00001	<0.00001	<0.00001	<0.00001	<0.00001	Ravn	Ravn
442362	<0.00001	1.00000	<0.00001	<0.00001	<0.00001	<0.00001	<0.00001	<0.00001	Ravn	Ravn
442422	<0.00001	1.00000	<0.00001	<0.00001	<0.00001	<0.00001	<0.00001	<0.00001	Ravn	Ravn
442508	<0.00001	1.00000	<0.00001	<0.00001	<0.00001	<0.00001	<0.00001	<0.00001	Ravn	Ravn
443118	<0.00001	1.00000	<0.00001	<0.00001	<0.00001	<0.00001	<0.00001	<0.00001	Ravn	Ravn
444147	<0.00001	1.00000	<0.00001	<0.00001	<0.00001	<0.00001	<0.00001	<0.00001	Ravn	Ravn
444593	0.00006	0.99994	<0.00001	<0.00001	<0.00001	<0.00001	<0.00001	<0.00001	Ravn	Ravn

Sample ID	Marburg	Ravn	Ebola	Bundibugyo	Taï Forest	Sudan	Reston	Negative	Assigned class	True class
550730	0.02364	0.97636	<0.00001	<0.00001	<0.00001	<0.00001	<0.00001	<0.00001	Ravn	Ravn
657147	<0.00001	1.00000	<0.00001	<0.00001	<0.00001	<0.00001	<0.00001	<0.00001	Ravn	Ravn
685137	0.00464	0.99536	<0.00001	<0.00001	<0.00001	<0.00001	<0.00001	<0.00001	Ravn	Ravn
686329	<0.00001	1.00000	<0.00001	<0.00001	<0.00001	<0.00001	<0.00001	<0.00001	Ravn	Ravn
214703	<0.00001	<0.00001	1.00000	<0.00001	<0.00001	<0.00001	<0.00001	<0.00001	Ebola	Ebola
214755	<0.00001	<0.00001	1.00000	<0.00001	<0.00001	<0.00001	<0.00001	<0.00001	Ebola	Ebola
214838	<0.00001	<0.00001	1.00000	<0.00001	<0.00001	<0.00001	<0.00001	<0.00001	Ebola	Ebola
220406	<0.00001	<0.00001	1.00000	<0.00001	<0.00001	<0.00001	<0.00001	<0.00001	Ebola	Ebola
369437	<0.00001	<0.00001	1.00000	<0.00001	<0.00001	<0.00001	<0.00001	<0.00001	Ebola	Ebola
421936	<0.00001	<0.00001	1.00000	<0.00001	<0.00001	<0.00001	<0.00001	<0.00001	Ebola	Ebola
441204	<0.00001	<0.00001	1.00000	<0.00001	<0.00001	<0.00001	<0.00001	<0.00001	Ebola	Ebola
442221	<0.00001	<0.00001	1.00000	<0.00001	<0.00001	<0.00001	<0.00001	<0.00001	Ebola	Ebola
444308	<0.00001	<0.00001	1.00000	<0.00001	<0.00001	<0.00001	<0.00001	<0.00001	Ebola	Ebola
444345	<0.00001	<0.00001	1.00000	<0.00001	<0.00001	<0.00001	<0.00001	<0.00001	Ebola	Ebola
546240	<0.00001	<0.00001	1.00000	<0.00001	<0.00001	<0.00001	<0.00001	<0.00001	Ebola	Ebola
547881	<0.00001	<0.00001	1.00000	<0.00001	<0.00001	<0.00001	<0.00001	<0.00001	Ebola	Ebola
652967	<0.00001	<0.00001	1.00000	<0.00001	<0.00001	<0.00001	<0.00001	<0.00001	Ebola	Ebola
656745	<0.00001	<0.00001	1.00000	<0.00001	<0.00001	<0.00001	<0.00001	<0.00001	Ebola	Ebola
686263	<0.00001	<0.00001	1.00000	<0.00001	<0.00001	<0.00001	<0.00001	<0.00001	Ebola	Ebola
691073	<0.00001	<0.00001	1.00000	<0.00001	<0.00001	<0.00001	<0.00001	<0.00001	Ebola	Ebola
720908	<0.00001	<0.00001	1.00000	<0.00001	<0.00001	<0.00001	<0.00001	<0.00001	Ebola	Ebola
721106	<0.00001	<0.00001	1.00000	<0.00001	<0.00001	<0.00001	<0.00001	<0.00001	Ebola	Ebola
721253	<0.00001	<0.00001	1.00000	<0.00001	<0.00001	<0.00001	<0.00001	<0.00001	Ebola	Ebola
726214	<0.00001	<0.00001	1.00000	<0.00001	<0.00001	<0.00001	<0.00001	<0.00001	Ebola	Ebola
193530	<0.00001	<0.00001	<0.00001	1.00000	<0.00001	<0.00001	<0.00001	<0.00001	Bundibugyo	Bundibugyo
267617	<0.00001	<0.00001	<0.00001	1.00000	<0.00001	<0.00001	<0.00001	<0.00001	Bundibugyo	Bundibugyo
268040	<0.00001	<0.00001	<0.00001	1.00000	<0.00001	<0.00001	<0.00001	<0.00001	Bundibugyo	Bundibugyo
268249	<0.00001	<0.00001	<0.00001	1.00000	<0.00001	<0.00001	<0.00001	<0.00001	Bundibugyo	Bundibugyo
283052	<0.00001	<0.00001	<0.00001	1.00000	<0.00001	<0.00001	<0.00001	<0.00001	Bundibugyo	Bundibugyo
283640	<0.00001	<0.00001	<0.00001	1.00000	<0.00001	<0.00001	<0.00001	<0.00001	Bundibugyo	Bundibugyo
283653	<0.00001	<0.00001	<0.00001	1.00000	<0.00001	<0.00001	<0.00001	<0.00001	Bundibugyo	Bundibugyo
284322	<0.00001	<0.00001	<0.00001	1.00000	<0.00001	<0.00001	<0.00001	<0.00001	Bundibugyo	Bundibugyo
284686	<0.00001	<0.00001	<0.00001	1.00000	<0.00001	<0.00001	<0.00001	<0.00001	Bundibugyo	Bundibugyo
285732	<0.00001	<0.00001	<0.00001	1.00000	<0.00001	<0.00001	<0.00001	<0.00001	Bundibugyo	Bundibugyo
287812	<0.00001	<0.00001	<0.00001	1.00000	<0.00001	<0.00001	<0.00001	<0.00001	Bundibugyo	Bundibugyo
289997	<0.00001	<0.00001	<0.00001	1.00000	<0.00001	<0.00001	<0.00001	<0.00001	Bundibugyo	Bundibugyo
290362	<0.00001	<0.00001	<0.00001	1.00000	<0.00001	<0.00001	<0.00001	<0.00001	Bundibugyo	Bundibugyo
290539	<0.00001	<0.00001	<0.00001	1.00000	<0.00001	<0.00001	<0.00001	<0.00001	Bundibugyo	Bundibugyo
290811	<0.00001	<0.00001	<0.00001	1.00000	<0.00001	<0.00001	<0.00001	<0.00001	Bundibugyo	Bundibugyo

Sample ID	Marburg	Ravn	Ebola	Bundibugyo	Taï Forest	Sudan	Reston	Negative	Assigned class	True class
266913	<0.00001	<0.00001	<0.00001	<0.00001	1.00000	<0.00001	<0.00001	<0.00001	Tai Forest	Tai Forest
267385	<0.00001	<0.00001	<0.00001	<0.00001	1.00000	<0.00001	<0.00001	<0.00001	Tai Forest	Tai Forest
268560	<0.00001	<0.00001	<0.00001	<0.00001	1.00000	<0.00001	<0.00001	<0.00001	Tai Forest	Tai Forest
283017	<0.00001	<0.00001	<0.00001	<0.00001	1.00000	<0.00001	<0.00001	<0.00001	Tai Forest	Tai Forest
283226	<0.00001	<0.00001	<0.00001	<0.00001	1.00000	<0.00001	<0.00001	<0.00001	Tai Forest	Tai Forest
283394	<0.00001	<0.00001	<0.00001	<0.00001	1.00000	<0.00001	<0.00001	<0.00001	Tai Forest	Tai Forest
283469	<0.00001	<0.00001	<0.00001	<0.00001	1.00000	<0.00001	<0.00001	<0.00001	Tai Forest	Tai Forest
283494	<0.00001	<0.00001	<0.00001	<0.00001	1.00000	<0.00001	<0.00001	<0.00001	Tai Forest	Tai Forest
283529	<0.00001	<0.00001	<0.00001	<0.00001	1.00000	<0.00001	<0.00001	<0.00001	Tai Forest	Tai Forest
284026	<0.00001	<0.00001	<0.00001	<0.00001	1.00000	<0.00001	<0.00001	<0.00001	Tai Forest	Tai Forest
284619	<0.00001	<0.00001	<0.00001	<0.00001	1.00000	<0.00001	<0.00001	<0.00001	Tai Forest	Tai Forest
285842	<0.00001	<0.00001	<0.00001	<0.00001	1.00000	<0.00001	<0.00001	<0.00001	Tai Forest	Tai Forest
287221	<0.00001	<0.00001	<0.00001	<0.00001	1.00000	<0.00001	<0.00001	<0.00001	Tai Forest	Tai Forest
290580	<0.00001	<0.00001	<0.00001	<0.00001	1.00000	<0.00001	<0.00001	<0.00001	Tai Forest	Tai Forest
926768	<0.00001	<0.00001	<0.00001	<0.00001	1.00000	<0.00001	<0.00001	<0.00001	Tai Forest	Tai Forest
215039	<0.00001	<0.00001	<0.00001	<0.00001	<0.00001	1.00000	<0.00001	<0.00001	Sudan	Sudan
434494	<0.00001	<0.00001	<0.00001	<0.00001	<0.00001	1.00000	<0.00001	<0.00001	Sudan	Sudan
436158	<0.00001	<0.00001	<0.00001	<0.00001	<0.00001	1.00000	<0.00001	<0.00001	Sudan	Sudan
437138	<0.00001	<0.00001	<0.00001	<0.00001	<0.00001	1.00000	<0.00001	<0.00001	Sudan	Sudan
439698	<0.00001	<0.00001	<0.00001	<0.00001	<0.00001	1.00000	<0.00001	<0.00001	Sudan	Sudan
441205	<0.00001	<0.00001	<0.00001	<0.00001	<0.00001	1.00000	<0.00001	<0.00001	Sudan	Sudan
441296	<0.00001	<0.00001	<0.00001	<0.00001	<0.00001	1.00000	<0.00001	<0.00001	Sudan	Sudan
441348	<0.00001	<0.00001	<0.00001	<0.00001	<0.00001	1.00000	<0.00001	<0.00001	Sudan	Sudan
441495	<0.00001	<0.00001	<0.00001	<0.00001	<0.00001	1.00000	<0.00001	<0.00001	Sudan	Sudan
441535	<0.00001	<0.00001	<0.00001	<0.00001	<0.00001	1.00000	<0.00001	<0.00001	Sudan	Sudan
441579	<0.00001	<0.00001	<0.00001	<0.00001	<0.00001	1.00000	<0.00001	<0.00001	Sudan	Sudan
441694	<0.00001	<0.00001	<0.00001	<0.00001	<0.00001	1.00000	<0.00001	<0.00001	Sudan	Sudan
442080	<0.00001	<0.00001	<0.00001	<0.00001	<0.00001	1.00000	<0.00001	<0.00001	Sudan	Sudan
443424	<0.00001	<0.00001	<0.00001	<0.00001	<0.00001	1.00000	<0.00001	<0.00001	Sudan	Sudan
443470	<0.00001	<0.00001	<0.00001	<0.00001	<0.00001	1.00000	<0.00001	<0.00001	Sudan	Sudan
556206	<0.00001	<0.00001	<0.00001	<0.00001	<0.00001	1.00000	<0.00001	<0.00001	Sudan	Sudan
647989	<0.00001	<0.00001	<0.00001	<0.00001	<0.00001	1.00000	<0.00001	<0.00001	Sudan	Sudan
657192	<0.00001	<0.00001	<0.00001	<0.00001	<0.00001	1.00000	<0.00001	<0.00001	Sudan	Sudan
720868	<0.00001	<0.00001	<0.00001	<0.00001	<0.00001	1.00000	<0.00001	<0.00001	Sudan	Sudan
284735	<0.00001	<0.00001	<0.00001	<0.00001	<0.00001	<0.00001	1.00000	<0.00001	Reston	Reston
286995	<0.00001	<0.00001	<0.00001	<0.00001	<0.00001	<0.00001	1.00000	<0.00001	Reston	Reston
289136	<0.00001	<0.00001	<0.00001	<0.00001	<0.00001	<0.00001	1.00000	<0.00001	Reston	Reston
289881	<0.00001	<0.00001	<0.00001	<0.00001	<0.00001	<0.00001	1.00000	<0.00001	Reston	Reston
422915	<0.00001	<0.00001	<0.00001	<0.00001	<0.00001	<0.00001	1.00000	<0.00001	Reston	Reston

Sample ID	Marburg	Ravn	Ebola	Bundibugyo	Taï Forest	Sudan	Reston	Negative	Assigned class	True class
550756	<0.00001	<0.00001	<0.00001	<0.00001	<0.00001	<0.00001	1.00000	<0.00001	Reston	Reston
637120	<0.00001	<0.00001	<0.00001	<0.00001	<0.00001	<0.00001	1.00000	<0.00001	Reston	Reston
690884	<0.00001	<0.00001	<0.00001	<0.00001	<0.00001	<0.00001	1.00000	<0.00001	Reston	Reston
691154	<0.00001	<0.00001	<0.00001	<0.00001	<0.00001	<0.00001	1.00000	<0.00001	Reston	Reston
691314	<0.00001	<0.00001	<0.00001	<0.00001	<0.00001	<0.00001	1.00000	<0.00001	Reston	Reston
691400	<0.00001	<0.00001	<0.00001	<0.00001	<0.00001	<0.00001	1.00000	<0.00001	Reston	Reston
691449	<0.00001	<0.00001	<0.00001	<0.00001	<0.00001	<0.00001	1.00000	<0.00001	Reston	Reston
720852	<0.00001	<0.00001	<0.00001	<0.00001	<0.00001	<0.00001	1.00000	<0.00001	Reston	Reston
721009	<0.00001	<0.00001	<0.00001	<0.00001	<0.00001	<0.00001	1.00000	<0.00001	Reston	Reston
724417	<0.00001	<0.00001	<0.00001	<0.00001	<0.00001	<0.00001	1.00000	<0.00001	Reston	Reston
26295	<0.00001	<0.00001	<0.00001	<0.00001	<0.00001	<0.00001	<0.00001	1.00000	Negative	Negative
34940	<0.00001	<0.00001	<0.00001	<0.00001	<0.00001	<0.00001	<0.00001	1.00000	Negative	Negative
41435	<0.00001	<0.00001	<0.00001	<0.00001	<0.00001	<0.00001	<0.00001	1.00000	Negative	Negative
41472	<0.00001	<0.00001	<0.00001	<0.00001	<0.00001	<0.00001	<0.00001	1.00000	Negative	Negative
85334	<0.00001	<0.00001	<0.00001	<0.00001	<0.00001	<0.00001	<0.00001	1.00000	Negative	Negative
86386	<0.00001	<0.00001	<0.00001	<0.00001	<0.00001	<0.00001	<0.00001	0.99999	Negative	Negative
91271	<0.00001	<0.00001	<0.00001	<0.00001	<0.00001	<0.00001	<0.00001	1.00000	Negative	Negative
214528	<0.00001	<0.00001	<0.00001	<0.00001	<0.00001	<0.00001	<0.00001	1.00000	Negative	Negative
442549	<0.00001	<0.00001	<0.00001	<0.00001	<0.00001	<0.00001	<0.00001	1.00000	Negative	Negative
547651	<0.00001	<0.00001	<0.00001	<0.00001	<0.00001	<0.00001	<0.00001	0.99999	Negative	Negative
550277	<0.00001	<0.00001	<0.00001	<0.00001	<0.00001	<0.00001	<0.00001	1.00000	Negative	Negative
644691	<0.00001	<0.00001	<0.00001	<0.00001	<0.00001	<0.00001	<0.00001	0.99999	Negative	Negative
651209	<0.00001	<0.00001	<0.00001	<0.00001	<0.00001	<0.00001	<0.00001	1.00000	Negative	Negative
653850	<0.00001	<0.00001	<0.00001	<0.00001	<0.00001	<0.00001	<0.00001	1.00000	Negative	Negative
684727	<0.00001	<0.00001	<0.00001	<0.00001	<0.00001	<0.00001	<0.00001	1.00000	Negative	Negative
685964	<0.00001	<0.00001	<0.00001	<0.00001	<0.00001	<0.00001	<0.00001	1.00000	Negative	Negative
725904	<0.00001	<0.00001	<0.00001	<0.00001	<0.00001	<0.00001	<0.00001	1.00000	Negative	Negative
726415	<0.00001	<0.00001	<0.00001	<0.00001	<0.00001	<0.00001	<0.00001	1.00000	Negative	Negative
742699	<0.00001	<0.00001	<0.00001	<0.00001	<0.00001	<0.00001	0.00002	0.99998	Negative	Negative

Supplementary Note. Serological data generated from a Marburg virus bat antiserum and a Ravn virus antiserum using our filovirus IgG indirect ELISA system was used to show how antibody fingerprinting was performed in this study. For simplicity, quadratic discriminate functions and posterior probability support values are shown for the inclusion of the two antisera in the Marburg and Ravn antigen classes only.

$x_{Antigen}$ is a vector listing the seven antigens in the filovirus IgG indirect ELISA system.

$$x_{Antigen} = [Marburg \quad Ravn \quad Ebola \quad Bundibugyo \quad Tai\,Forest \quad Sudan \quad Reston]$$

x_{443629} is a vector containing adjusted sum OD values for the reactivity between Marburg virus antiserum 443629 and the antigens listed in $x_{Antigen}$.

$$x_{443629} = [1.62 \quad 2.82 \quad 0.57 \quad -0.16 \quad 0.49 \quad -0.15 \quad 0.32]$$

x_{441344} is a vector containing adjusted sum OD values for the reactivity between Ravn virus antiserum 441344 and the antigens listed in $x_{Antigen}$.

$$x_{441344} = [1.5 \quad 2.5 \quad -0.28 \quad 0.11 \quad 0.71 \quad -0.004 \quad 0.425]$$

$\bar{x}_{Marburg}$ is a vector containing within class mean adjusted sum OD values for the reactivity between the Marburg virus antisera group (n=20) and the antigens listed in $x_{Antigen}$.

$$\bar{x}_{Marburg} = [3.43 \quad 3.99 \quad 0.246 \quad 0.465 \quad 0.419 \quad -0.4 \quad 0.212]$$

$\bar{x}_{Ravn}$ is a vector containing within class mean adjusted sum OD values for the reactivity between the Ravn virus antisera group (n=20) and the antigens listed in $x_{Antigen}$.

$$\bar{x}_{Ravn} = [2.98 \quad 4.86 \quad -0.07 \quad 0.166 \quad 0.428 \quad 0.046 \quad 0.341]$$

$\hat{\Sigma}_{Marburg}$ is a symmetric within class covariance matrix with rows denoted by $x_{Antigen}$ and columns denoted by $x_{Antigen}$. The diagonal entries represent the variance in adjusted sum OD values for the reactivity of the Marburg virus antisera group (n=20) with each of the seven antigens, while the remaining entries represent the covariance in adjusted sum OD values for the reactivity of the Marburg virus antisera group with two antigens. For example, $\hat{\Sigma}_{Marburg1,2}$ represents the covariance in adjusted sum OD values for the reactivity of the Marburg virus antisera group with Marburg and Ravn virus antigens.

$$\hat{\Sigma}_{Marburg} = \begin{bmatrix} 3.14 & 3.47 & 0.06 & -0.06 & -0.16 & 0.15 & -0.07 \\ 3.47 & 4.28 & 0.03 & -0.1 & -0.18 & 0.14 & -0.09 \\ 0.06 & 0.03 & 0.05 & 0.01 & 0.02 & 0.05 & 0.02 \\ -0.06 & -0.1 & 0.01 & 0.03 & 0.02 & 0.01 & 0.01 \\ -0.16 & -0.18 & 0.02 & 0.02 & 0.03 & 0.01 & 0.02 \\ 0.15 & 0.14 & 0.05 & 0.01 & 0.01 & 0.02 & 0.02 \\ -0.07 & -0.09 & 0.02 & 0.01 & 0.02 & 0.02 & 0.2 \end{bmatrix}$$

$\hat{\Sigma}_{Ravn}$ is a symmetric within class covariance matrix with rows denoted by $x_{Antigen}$ and columns denoted by $x_{Antigen}$. The diagonal entries represent the variance in adjusted sum OD values for the

reactivity of the Ravn virus antisera group (n=20) with each of the seven antigens, while the remaining entries represent the covariance in adjusted sum OD values for the reactivity of the Ravn virus antisera group with two antigens.

$$\hat{\Sigma}_{Ravn} = \begin{bmatrix} 1.89 & 1.83 & 0.03 & 0.12 & 0.13 & 0.06 & 0.02 \\ 1.83 & 2.44 & 0.03 & 0.11 & 0.13 & 0.04 & 0.03 \\ 0.03 & 0.03 & 0.03 & 0.02 & 0.01 & 0.02 & -0.001 \\ 0.12 & 0.11 & 0.02 & 0.04 & 0.03 & 0.01 & 0.01 \\ 0.13 & 0.13 & 0.01 & 0.03 & 0.05 & 0.01 & 0.01 \\ 0.06 & 0.04 & 0.02 & 0.01 & 0.01 & 0.03 & 0.003 \\ 0.02 & 0.03 & -0.001 & 0.01 & 0.01 & 0.003 & 0.02 \end{bmatrix}$$

$dQ_{Marburg}(x)$ is the quadratic discriminant function used to calculate likelihood values for inclusion of Marburg virus antiserum 443629 and Ravn antiserum 441344 in the Marburg virus antigen class. Similar quadratic discriminant functions are used to calculate likelihood values for inclusion of these two bat antisera in the Ravn antigen class and other filovirus antigen classes.

$$dQ_{Marburg}(x) = -\frac{1}{2} ln\left(\left|\hat{\Sigma}_{Marburg}\right|\right) - \frac{1}{2}\left(x - \bar{x}_{Marburg}\right)^T \hat{\Sigma}_{Marburg}^{-1}\left(x - \bar{x}_{Marburg}\right) + ln\left(p_{Marburg}\right)$$

$$dQ_{Marburg}(443629) = 2.316$$

$$dQ_{Ravn}(443629) = -16.22$$

$$dQ_{Marburg}(441344) = -54.84$$

$$dQ_{Ravn}(441344) = 4.003$$

Classification is performed by selecting the quadratic discriminant score that is the highest for each antiserum. The quadratic discriminant scores can also be used to calculate posterior probabilities that can be used to predict which of the filovirus species in our filovirus IgG indirect ELISA system is the most antigenically similar to the species responsible for past infection, essentially creating an antibody fingerprint. $pp_{Marburg}(x)$ was used to calculate the posterior probability for inclusion of Marburg virus antiserum 443629 and Ravn antiserum 441344 in the Marburg class.

$$pp_{Marburg}(x) = \frac{e^{dQ_{Marburg}(x)}}{\sum_{i=1}^{C} e^{dQ_i(x)}}$$

Below are posterior probability values for the inclusion of Marburg virus antiserum 443629 and Ravn antiserum 441344 in the Marburg and Ravn classes. Although not shown here, the posterior probabilities for the inclusion of Marburg virus antiserum 443629 and Ravn antiserum 441344 in the Ebola, Bundibugyo, Taï Forest, Sudan, Reston and Negative classes were < 0.00001.

$$pp_{Marburg}(443629) = 1.00000$$

$$pp_{RAVN}(443629) < 0.00001$$

$$pp_{Marburg}(441344) < 0.00001$$

$$pp_{Marburg}(441344) = 1.00000$$

Based on these posterior probabilities, bats 443629 and 441344 were most likely to have been previously infected with a virus within the species *Marburg marburgvirus* (e.g., Marburg, Ravn or an undiscovered virus belonging to this species).
